# Cardiac magnetic resonance imaging in patients referred for premature ventricular contractions: identifying patients most likely to benefit

**DOI:** 10.3389/fcvm.2026.1875202

**Published:** 2026-07-01

**Authors:** Amélie Gomez, Mesut Gun, Akli Otmani, Laurent Leborgne, Christophe Tribouilloy, Emmanuelle Vermes

**Affiliations:** 1 Department of Cardiology, Amiens University Medical Center, Amiens, France; 2 UR UPJV 7517, Jules Verne University of Picardie, Amiens, France

**Keywords:** cardiac magnetic resonance, cardiomyopathy, diabetes, ischemic fibrosis, nonischemic fibrosis, premature ventricular contraction

## Abstract

**Background:**

The value of cardiac magnetic resonance (CMR) imaging in cases of premature ventricular contractions (PVCs) has not been clearly defined.

**Objectives:**

To assess the ability of CMR to detect structural abnormalities in patients referred for PVCs and to determine predictive factors.

**Methods:**

200 consecutive patients were retrospectively included after a workup that included an electrocardiogram, echocardiography, and an exercise test. We determined whether or not CMR could reveal previously undetected structural abnormalities.

**Results:**

CMR revealed structural abnormalities in 92 patients (46% of cases): nonischemic fibrosis (*n* = 53, 26.5%), ischemic fibrosis (*n* = 18, 9%), mixed fibrosis (*n* = 4, 2%), and 4 (2%) cardiomyopathies. In a multivariate analysis, the main predictors of structural abnormalities were age [odds ratio (OR): 1.03; *p* = 0.016], male sex (OR: 2.48; *p* = 0.019), diabetes (OR: 5.02; *p* = 0.010), smoking (OR: 2.09; *p* = 0.047), known cardiomyopathy (OR: 3.24; *p* = 0.006), pleomorphic PVCs (OR: 2.48; *p* = 0.051), and the absence of a decrease in PVC frequency during exercise (OR: 0.24; *p* < 0.001). In contrast, all young, nondiabetic subjects with unknown cardiomyopathy, normal echocardiography, and monomorphic PVC that decreased in frequency with effort had normal CMR findings.

**Conclusions:**

CMR is essential in the investigation of PVCs; it enabled the detection of structural abnormalities not found during the initial workup in 46% of the cases. Predictive factors included male sex, age, diabetes, smoking, known cardiomyopathy, and pleomorphic PVCs. In contrast, young and nondiabetic patients with normal echocardiography and monomorphic PVCs that decrease in frequency with effort had normal CMR findings; raising the hypothesis that the usefulness of CMR in this specific population should be discussed.

## Introduction

Premature ventricular contractions (PVCs), symptomatic or discovered incidentally on an electrocardiogram (ECG), are common in the general population and considered to be benign, with the patient's prognosis is similar to that of the general population (1). In a subgroup of patients, however, PVCs can indicate the presence of structural heart disease (SHD), essential to detect in order to mitigate the risk of malignant ventricular arrhythmia and sudden cardiac death (SCD). According to the latest guidelines (2), the initial assessment of PVCs should include an ambulatory 24 h ECG and transthoracic echocardiography (TTE). However, TTE does not always enable the evaluation of right ventricle (RV) morphology, and myocardial fibrosis (a powerful predictor of ventricular arrhythmia) cannot be assessed.

Cardiac magnetic resonance (CMR) is a noninvasive modality with dynamic sequences particularly useful for evaluating the RV, with clear visualization of the infundibulum, pulmonary valve, and RV outflow tract. Furthermore, Late gadolinium enhancement (LGE) sequences can detect focal myocardial fibrosis (3). However, the status of CMR in clinical guidelines is unclear. CMR is a valuable adjunct to the initial evaluation when the initial workup does not exclude underlying SHD or when the patient has an atypical clinical presentation, such as advanced age (although a cut-off is not mentioned) or right bundle branch block (suggesting a left ventricular origin) (2). In clinical practice, however, cardiologists frequently request CMR due to its ability to detect structural abnormalities not visible with TTE. However, most studies included patients with a normal initial assessment (4), which does not correspond to the general patient population in a university medical center setting.

The primary objective of this study was to evaluate the performance of CMR to detect structural abnormalities in patients referred for PVCs by their cardiologist without any restrictions. The secondary objective was to determine which clinical, imaging and laboratory factors might be predictive of the presence or absence of structural cardiac abnormalities and might therefore obviate the need for CMR.

## Materials and methods

### Study population

Adult patients referred by cardiologists to Amiens University Medical Center (France) for CMR with an indication of “PVCs or ventricular hyperexcitability” between May 2022 and April 2024 were retrospectively and consecutively included. Ventricular hyperexcitability can be defined by any ventricular arrhythmia (PVCs, sustained or nonsustained ventricular tachycardia). Only patients with an unknown PVC morphology or poor image quality were excluded.

The protocol conforms to the ethical guidelines of the 1975 Declaration of Helsinki as reflected in *a priori* approval by the hospital research committee and registered at ClinicalTrials.gov (NCT06801743) and under the institutional research identifier PI2023_843_0169 (IRMESV).

### Data collection

Clinical and laboratory data were collected from the patients’ electronic medical records. We chose to classify pre-existing mitral valve prolapse separately from other valve diseases, due to the known arrhythmogenic risk (5). The PVC morphology on a 12-lead ECG was classified into one of four categories. The 24-hour ambulatory ECG recordings were classified into one of three categories, depending on the frequency of PVCs (<1000 PVCs/24 h; 1000–10 000 PVCs/24 h; or ≥ 10000 PVCs/24 h).

### CMR acquisitions

CMR examinations were performed using a 1.5 Tesla magnet (GE Artist 30.1) and 3 T Philips (Achieva, dStream 5.7). LV and RV function as well as wall ventricular motion were assessed using standard ECG-gated cine steady state free precession sequences [typical repetition time (TR) = 67, echo delay time = 1.15, field of view 340 × 276]. Standard short axis, long axis cine images including 2, 3, and 4 chamber views were acquired as well as 2 specialized views for the RV: vertical long axis and sagittal orientation. Late gadolinium enhancement (LGE) images were obtained by use of phase sensitivity inversion recovery technique (PSIR) with slice thickness 8 mm, gap 1.5 mm for short axis, and 3D PSIR with slice thickness 5 mm with no interslice gap for 2,3 and 4 chamber-views, TR set to the patient's heart rate, field of view 340–400 mm. LGE sequences were acquired 10 min after injection of gadolinium. Subendocardial and transmural LGE in a coronary segment were considered ischemic fibrosis. Subepicardial, mid-parietal and right ventricular insertion points LGE were considered non-ischemic fibrosis. T1/T2 mapping were not performed. RV abnormalities were defined as a dilatation and/or systolic dysfunction or a wall motion abnormality (hypokinesia, akinesia or dyskinesia). All CMRs were reviewed retrospectively by the same cardiologist blinded to clinical, biological and echocardiographic data and used specific, certified software (Circle™ version 5.16.0). No major differences between 1.5 and 3 T regarding wall motion abnormalities or focal fibrosis assessment were noticed by the cardiologist.

### Study outcomes

The primary outcome was the ability of CMR to identify SHD not detected with the initial workup.

The secondary outcome was the identification of predictive factors for the presence or absence of structural abnormalities on CMR, in order to patient profiles for which this examination could be avoided.

### Statistical analysis

Continuous variables were reported as the mean ± standard deviation (SD) for normally distributed data and the median [interquartile range (IQR)] for nonnormally distributed data. Student's t-test was used to compare normally distributed continuous variables between groups, while the Mann–Whitney (or Wilcoxon) test was used for nonnormally distributed data. For comparisons involving more than two groups, an analysis of variance and the Kruskal–Wallis test were applied for normally distributed data and nonnormally distributed data, respectively. Categorical variables were compared using the chi-squared (*χ*^2^) test or (if at least one contingency table cell had a count below 5) Fisher's exact test. The results were expressed as mean differences or relative risks, depending on the type of variable.

In a multivariate logistic regression analysis, the glm function and the binomial family in R software were used to identify independent predictors. The variables presented in Table 1 with a *p*-value below 0.10 were considered for initial inclusion. Multicollinearity was assessed using the variance inflation factor; variables with a factor greater than 5 were removed. Although a larger number of candidate variables were initially explored, a stepwise reduction process was applied to obtain a parsimonious model, and the final multivariable model retained a limited number of predictors in order to reduce the risk of overfitting and ensure model stability. The results were expressed as odds ratios (ORs).

**Table 1:** Univariate analysis of clinical, TTE, ECG and functional testing characteristics, according to the CMR findings.

Characteristic	Overall (*N* = 200)	No CMR anomaly (*N* = 108)	CMR structural anomaly (*N* = 92)	*p*-value
Age (mean, SD)	59 (15)	55 (16)	64 (11)	<0.001
Sex				<0.001
Females (%)	71 (36%)	52 (48%)	19 (21%)	
Males (%)	129 (65%)	56 (52%)	73 (79%)	
BMI (mean, SD)	26.7 (4.8)	26.0 (5.0)	27.4 (4.6)	0.041
Diabetes (%)	25 (13%)	4 (3.7%)	21 (23%)	<0.001
Dyslipidemia (%)	47 (24%)	12 (11%)	35 (38%)	<0.001
Hypertension (%)	83 (42%)	37 (34%)	46 (50%)	0.024
Smoking (%)	70 (35%)	31 (29%)	39 (42%)	0.043
Atrial arrhythmia (AF or flutter) (%)	27 (14%)	7 (6.5%)	20 (22%)	0.002
Known cardiomyopathy (%)	46 (23%)	13 (12%)	33 (36%)	<0.001
Palpitations (%)	72 (36%)	47 (44%)	25 (27%)	0.016
Syncope (%)	22 (11%)	8 (7.4%)	14 (15%)	0.079
LBBB, positive in the inferior leads, PVC (%)	70 (35%)	47 (44%)	23 (25%)	0.006
RBBB, negative in inferior leads, PVC (%)	59 (30%)	26 (24%)	33 (36%)	0.068
Pleiomorphic PVC (%)	38 (19%)	12 (11%)	26 (28%)	0.002
NSVT (%)	64 (32%)	23 (21%)	41 (45%)	<0.001
SVT (%)	5 (2.5%)	1 (0.9%)	4 (4.3%)	0.2
TTE anomaly (%)	64 (32%)	23 (21%)	41 (45%)	<0.001
Increase in PVC frequency with exercise (%)	67 (34%)	27 (25%)	40 (43%)	0.006
Decrease in PVC frequency with exercise (%)	76 (38%)	59 (55%)	17 (18%)	<0.001

The data are expressed as the mean (SD) or *n* (%).

BMI, body mass index; AF, atrial fibrillation; LBBB, left bundle branch block; RBBB, right bundle branch block; PVC, premature ventricular contraction; NSVT, nonsustained ventricular tachycardia; SVT, sustained ventricular tachycardia; TTE, transthoracic echocardiography.

A receiver operating characteristic (ROC) curve analysis was performed on the final multivariable logistic regression model developed to predict the presence of structural abnormalities detected by CMR. The Youden index was used to determine the optimal threshold for identifying patients at risk. An exploratory analysis was then conducted using age categories defined according to quartiles of the study population to assess the impact of age on model performance. Sensitivity (Se) and specificity (Sp) were calculated and the optimal cutoff was identified.

A complementary analysis was performed by using the cut function in R software to divide the population into four age groups (i.e., quartiles of age, as a continuous variable). Through the application of an adjusted logistic regression model, this analysis explored the effects of age on CMR referral.

An additional analysis compared patients with a normal CMR result vs. those with abnormalities. A V-test analysis was performed: a bar chart represented the strength of the associations between a normal CMR result and clinical covariates.

To address the missing data, a k-nearest neighbors (kNN) imputation method was applied. This nonparametric approach estimates missing values from the similarity of observations in the space of available variables. This imputation was performed using the kNN function from the *VIM* package in R, with the k parameter set to 5 neighbors. The robustness and validity of kNN imputation have been demonstrated in a number of studies of complex biological data (6). Variables with missing data were imputed simultaneously, without the creation of indicator variables for imputation.

All analyses were performed using R software (version 4.3.2; R Core Team, 2025; R Foundation for Statistical Computing, Vienna, Austria).

## Results

### Clinical characteristics of the study population

**Figure 1:**
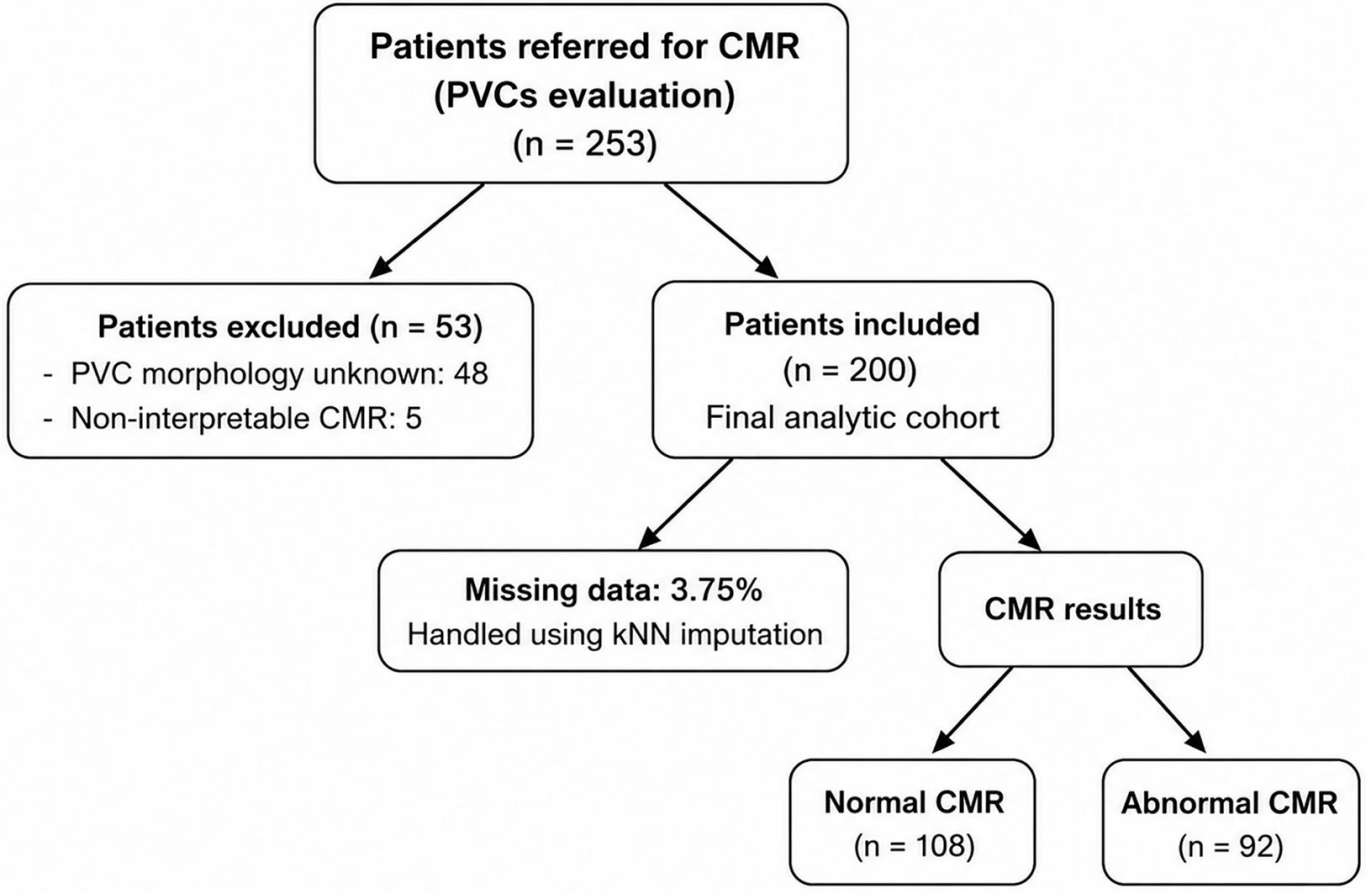
Flowchart of patient selection and study population. Of 253 patients referred for CMR for PVC evaluation, 53 were excluded. The final analytic cohort included 200 patients, among whom 108 had normal CMR findings and 92 had abnormal CMR findings. Missing data (3.75%) were handled using k-nearest neighbor (kNN) imputation.

**Figure 2:**
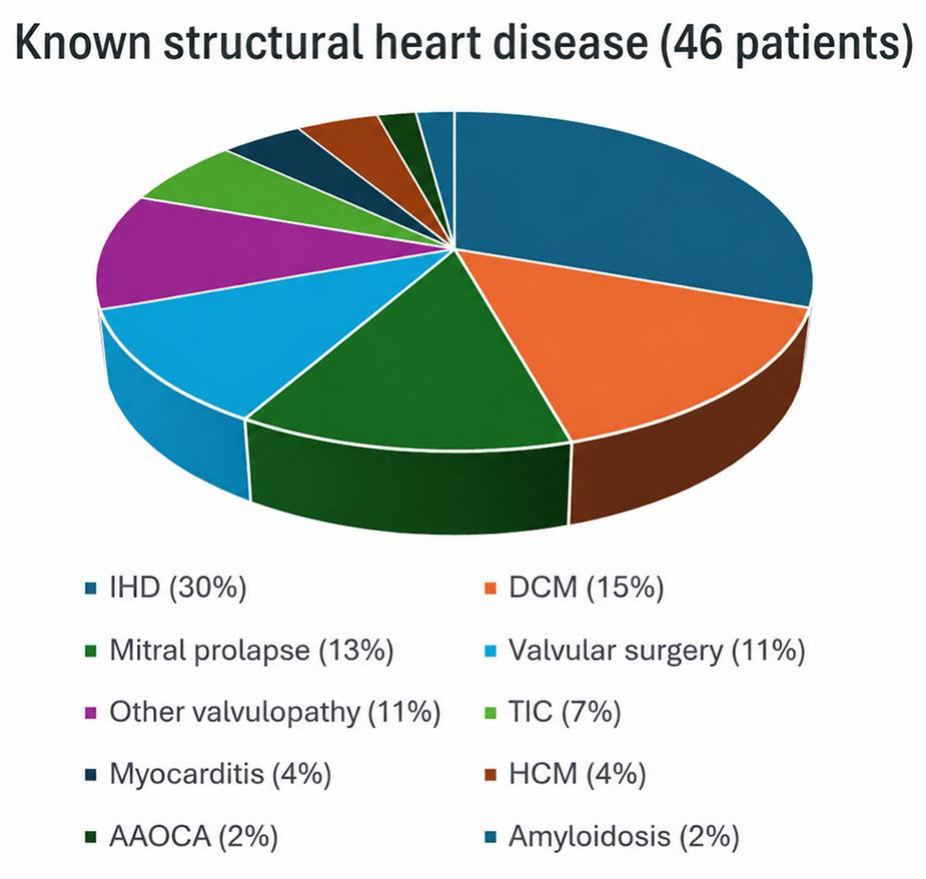
Known structural heart diseases before CMR. IHD, ischemic heart disease; DCM, dilated cardiomyopathy; TIC, tachycardia-induced cardiomyopathy; HCM, hypertrophic cardiomyopathy; AAOCA, anomalous aortic origin of a coronary artery.

Fifty-three patients were excluded. The study population comprised 200 patients (Figure 1). The mean age was 59 years, with more men (Table 2). Twenty-seven patients (14%) had a history of supraventricular arrhythmia, and 6 (3%) had a family history of SCD. The majority of patients were symptomatic (*n* = 126, 63%). 46 patients (23%) had a known structural heart disease (SHD), which was predominantly ischemic heart disease (*n* = 14, 30%), 7 dilated cardiomyopathy (DCM), 6 mitral prolapse, 5 valvular surgery, 5 other valvulopathy, 3 tachycardia-induced cardiomyopathy (TIC), 2 hypertrophic cardiomyopathy (HCM), 1 anomalous aortic origin of a coronary artery (AAOCA), 1 amyloidosis (Figure 2).

**Table 2:** Clinical characteristics of the study population.

Clinical characteristics	Total: *n* = 200
Age (years)	59 ± 15
Male sex	129 (65%)
Body mass index (kg/m^2^)	26.7 ± 4,8
Diabetes	25 (13%)
Dyslipidemia	47 (24%)
Hypertension	83 (42%)
Smoking	70 (35%)
History of atrial fibrillation or flutter	27 (14%)
Structural heart disease	46 (23%)
Family history of heart disease	17 (8,5%)
Family history of sudden death	6 (3%)
Symptoms None Chest pain Dyspnea Palpitations Dizziness Syncope	73 (37%) 25 (13%) 32 (16%) 72 (36%) 22 (11%) 12 (6%)

### Functional, TTE, ECG and coronary angiography characteristics of the study population

The main PVC morphology was LBBB and positive in the inferior leads (*n* = 70, 35%) (Table 3). 38 patients (19%) had PPVCs. The frequency of PVCs on ambulatory ECG was similar in the three groups. Non-sustained ventricular tachycardia (NSVT) was found in 64 (32%) patients and sustained ventricular tachycardia (SVT) in five (2.5%). The majority of the patients (*n* = 136, 68%) did not have significant TTE abnormalities. 127 patients performed an exercise test: PVCs increased with exercise in 44 (35%) cases and decreased with exercise in 48 (38%). Coronary angiography was performed in 43 patients and revealed abnormalities in 18 of them. It should be borne in mind that 14 study participants already had known ischemic heart disease, and so only four patients were newly diagnosed with coronary artery disease.

**Table 3:** ECG, TTE, exercise test and coronary angiography characteristics of the study population.

Predominant PVC pattern:	*n*/200 (%)
LBBB, positive in the inferior leads LBBB, negative in the inferior leads RBBB, positive in the inferior leads RBBB, negative in the inferior leads	70/200 (35%) 32/200 (16%) 39/200 (19,5%) 59/200 (29.5%)
Pleomorphic PVCs	38/200 (19%)
24 h ambulatory ECG	*n*/200 (%)
<1000 PVCs/24 h 1000–10000 PVCs/24 h >10000 PVCs/24 h	58/200 (38%) 67/200 (33.5%) 57/200 (28.5%)
NSVT	63/200 (32%)
SVT	5/200 (2.5%)
ECG abnormalities	*n*/200 (%)
Low-grade atrioventricular block High degree atrioventricular block Complete LBBB Complete RBBB Q-wave Inverted T waves Left ventricular hypertrophy Prolonged QTc interval None of the above	16/200 (8%) 1/200 (0.5%) 8/200 (4%) 15/200 (7.5%) 7/200 (3.5%) 18/200 (9%) 7/200 (3.5%) 5/200 (2.5%) 148/200 (74%)
TTE:	*n*/200 (%)
No significant abnormalitie LV dilatation LV kinetic anomaly LVEF <50% RV dilatation	136/200 (68%) 23/200 (12%) 19/200 (9.5%) 18/200 (9%) 13/200 (6.5%)
Exercise test:	*n*/127 (%)
Increase in PVC frequency during exercise Decrease in PVC frequency during exercise Increase in PVC frequency during recovery Positive for ischemia	44/127 (35%) 48/127 (38%) 30/127 (24%) 3/127 (2%)
Coronary angiography:	*n*/43 (%)
Coronary artery lesions No significant lesions	18/43 (42%) 25/43 (58%)

PVC, premature ventricular contractions; LBBB, left bundle branch block; RBBB, right bundle branch block; ECG, electrocardiogram; NSVT, nonsustained ventricular tachycardia; SVT, sustained ventricular tachycardia; TTE, transthoracic echocardiography; LV, left ventricle; LVEF, left ventricular ejection fraction; RV, right ventricular.

### CMR findings

Of the 200 patients, 92 (46%) had structural abnormalities. The most frequent finding (*n* = 53, 26.5%) was nonischemic fibrosis, which might have corresponded to a myocarditis scar in 17 of these cases. Isolated ischemic fibrosis was found in 18 (9%) patients and mostly corresponded to nonviable necrosis. Four patients had a combination of mixed ischemic and nonischemic fibrosis.

**Figure 3:**
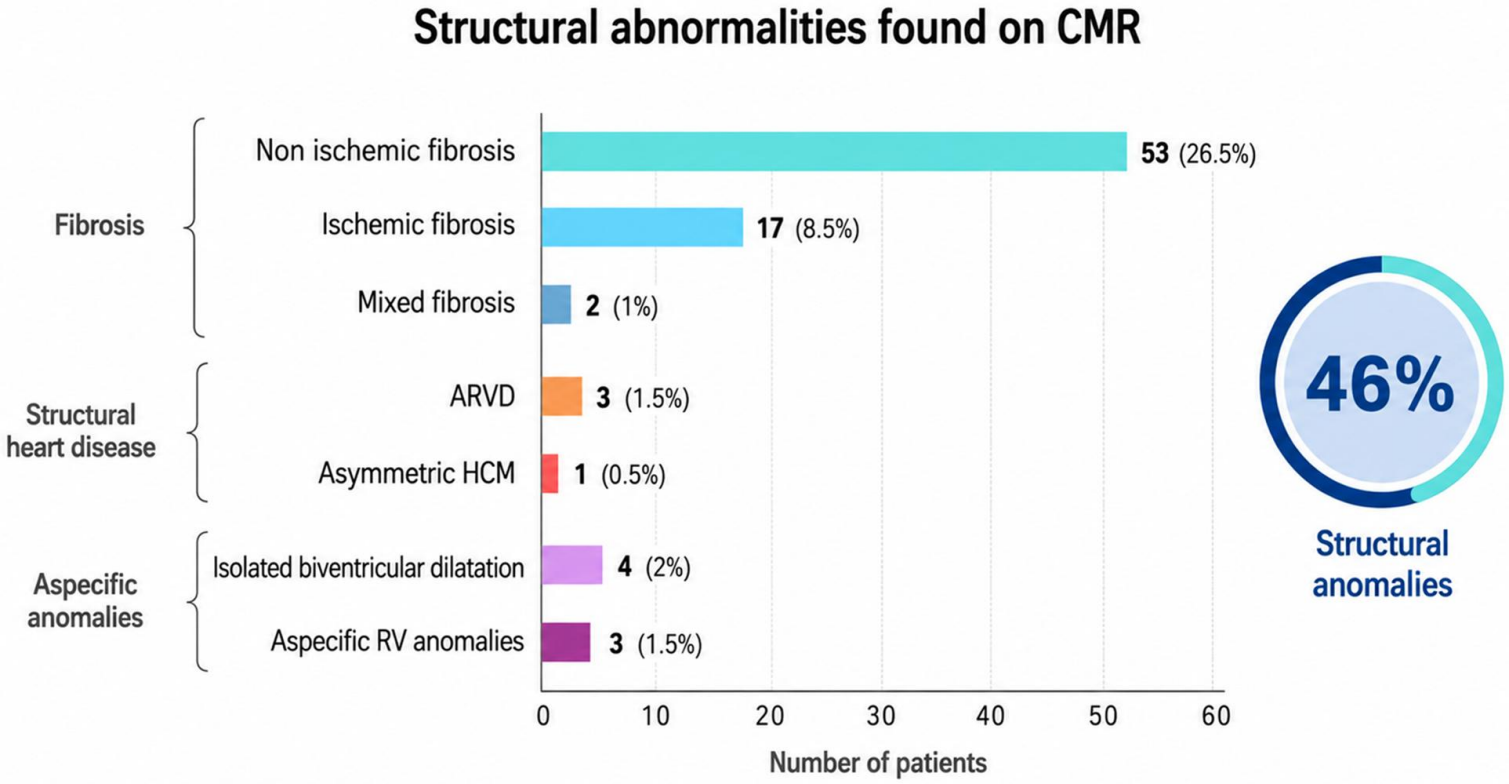
Structural abnormalities found on CMR. ARVD, arrhythmogenic right ventricular dysplasia; HCM, hypertrophic cardiomyopathy; RV, right ventricle; LV, left ventricle.

Four cardiomyopathies were identified: one case of HCM and three suspected cases of ARVD, according to the European Task Force consensus criteria (7). Of the 46 patients with known SHD, 17 (37%) had nonischemic fibrosis, and 13 (28%) had ischemic fibrosis (Figure 3 and Table 4). There were also two cases of mixed fibrosis.

**Table 4:** CMR results.

Quantitative variables	Mean (SD)
LVEDV (mL/m^2^)	82 (22)
LVEF (%)	57 (8)
LV mass (g/m^2^)	54 (13)
IVS (mm)	10.75 (2.28)
RVEDV (mL/m^2^)	79 (19)
RVEF (%)	51 (7)
Late gadolinium enhancement	75/200 (37.5%)
Non-ischemic pattern	53/200 (26.5%)
Non-viable necrosis	11/200 (5.5%)
Viable necrosis	6/200 (3.0%)
Mixed fibrosis	4/200 (2%)
Mixture of nonviable and viable necrosis	1/200 (0.5%)

LVEDV, left ventricular end-diastolic volume; LVEF, left ventricular ejection fraction; LV, left ventricle; IVS, interventricular septum; RVEDV, right ventricular end-diastolic volume.

We also identified a small number of minor nonspecific abnormalities, including four cases of isolated biventricular dilatation and three nonspecific RV abnormalities not matching all Task Force criteria for ARVD (one isolated RV dilatation, one isolated RV dysfunction and one with RV dilatation and dysfunction).

### Univariate and multivariate analyses of variables associated with normal or abnormal CMR findings

**Figure 4:**
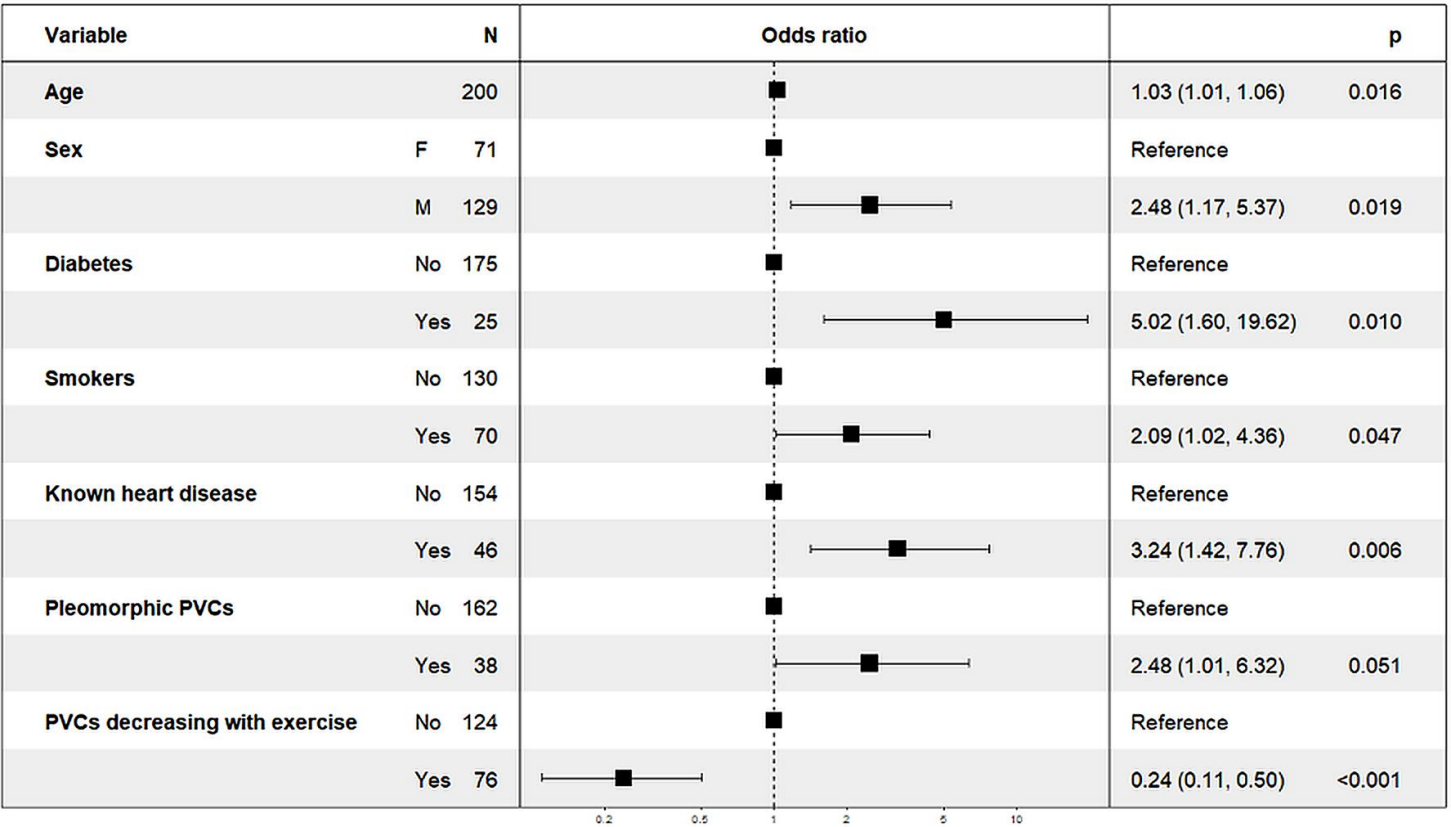
Multivariate analysis incorporating the variables significantly associated with abnormalities on cardiac magnetic resonance (CMR) with a *p*-value < 0.10. PVC, premature ventricular contraction.

**Figure 5:**
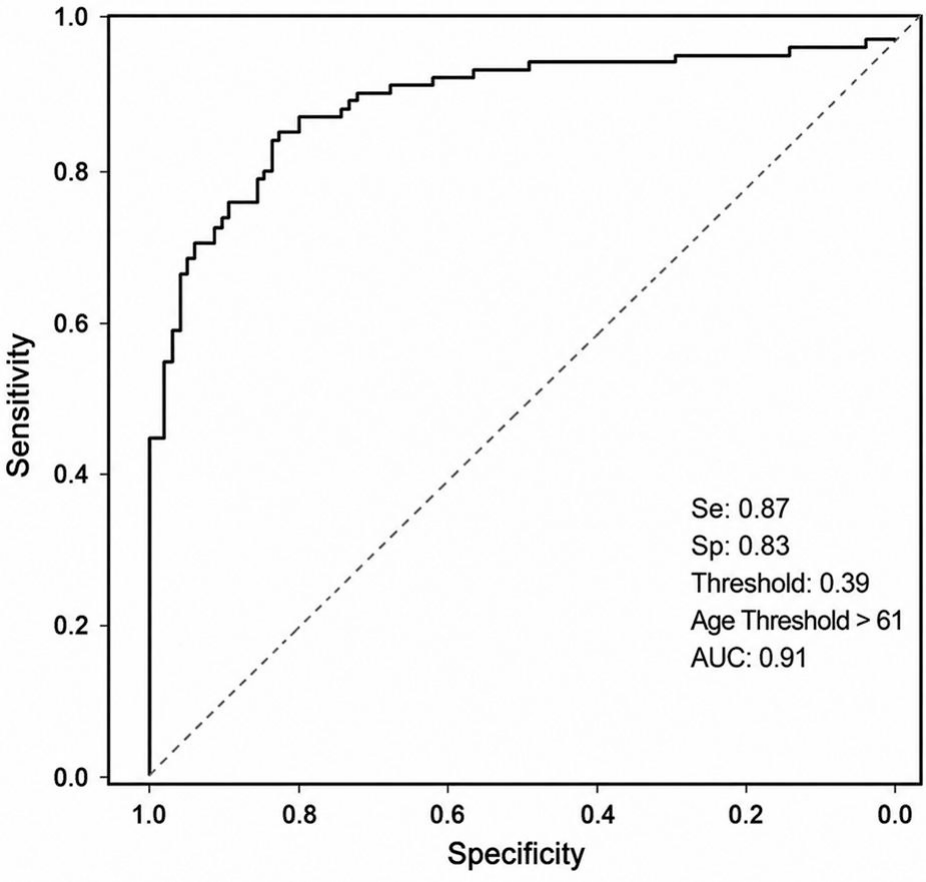
The receiver operating characteristic (ROC) curve, and the area under the curve (AUC) enabling discrimination between patients with structural abnormalities and those without. Sensitivity (Se) and specificity (Sp) were calculated for different probability thresholds, and the optimal threshold was identified.

**Figure 6:**
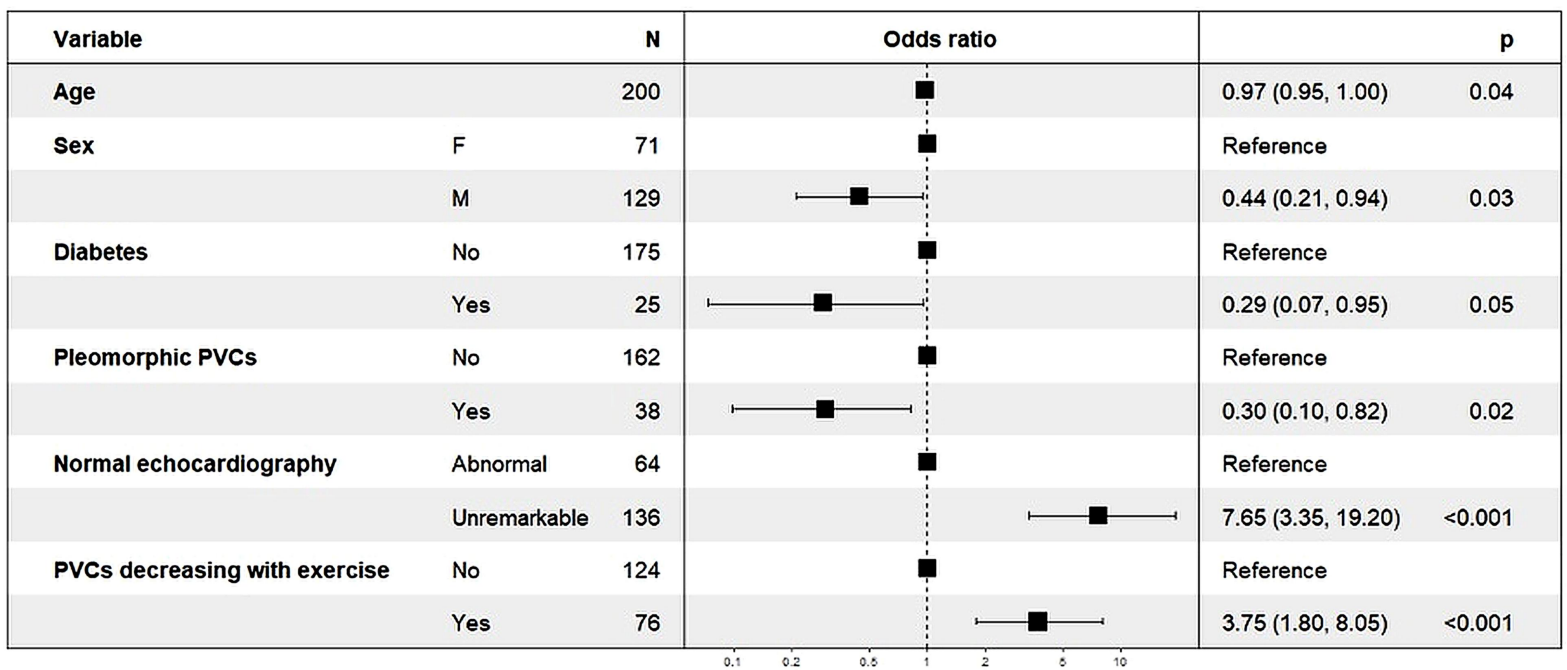
Factors predictive of normal CMR findings in a multivariate analysis. N, number; P, *p*-value; PVC, premature ventricular contraction.

In a univariate analysis, 16 variables were significantly associated with newly identified structural abnormalities on CMR (Table 1). In a multivariate analysis, seven of these variables were found to be independent predictors of CMR abnormalities (Figure 4). We were able to define an age threshold (> 61) significantly associated with CMR abnormalities, with a sensitivity of 87% and a specificity of 83% (Figure 5). In a multivariate analysis, we identified the factors significantly associated with normal CMR (Figure 6).

The overall proportion of missing data was low (3.70%). The majority of clinical, ECG, echocardiographic, and CMR variables were complete (0%). The main missing data points concerned coronary angiography (76.5%), stress test parameters (36.0–36.5%), and Holter ECG (14.0–18.0%), while late gadolinium enhancement variables on MRI had a low proportion of missing data (0.5%–8.5%). Imputation using the k-nearest neighbors (kNN) method was applied.

**Figure 7:**
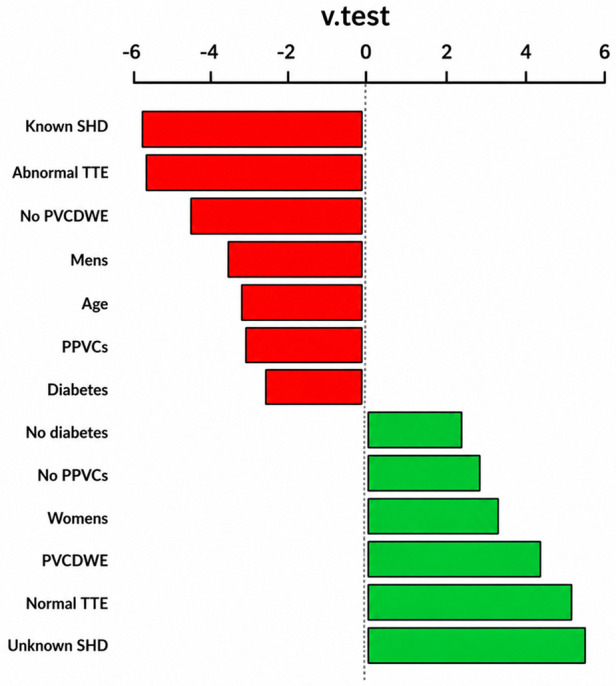
Bar chart representing covariates predictive of normal or abnormal CMR findings. Green bars represent the variables predicting normal CMR findings, and red bars represent the variables predicting abnormal CMR findings. The V-test represents the strength of the association between the various covariates and normal CMR findings. SHD, structural heart disease; TTE, transthoracic echocardiography; PVCDWE, premature ventricular contractions decreasing with exercise; PPVC, pleomorphic premature ventricular contractions.

In Figure 7, the variables in the green bar chart can be considered to be “green flags” suggestive of a normal CMR, while the variables in the red chart are “red flags” suggestive of a CMR anomaly.

## Discussion

### Main results

In our assessment of CMR for the evaluation of PVCs in the population referred by cardiologists, this imaging modality detected structural abnormalities in 46% of the patients, including 36% with known SHD.

To the best of our knowledge, the present study is the first to have analyzed the contribution of CMR, regardless of the patient's medical history or any other evaluations performed. Our study population corresponded to that encountered in routine practice with patients who may already have a history or clinical or paraclinical abnormalities suggesting a SHD and for whom CMR provided new information.

Most studies of CMR and PVCs included patients with normal results in the initial workup. A recent study of patients with at least 5% of PVCs on a 24-hour ECG but with normal TTE findings found that 35 (13.7%) of the 255 participants had structural abnormalities on CMR and that 28 (11%) showed LGE (8). A study of patients with ventricular arrhythmia showed that after a workup including echocardiography and coronary angiography, CMR revealed, modified or clarified the diagnosis of SHD in 40% of cases (9). A very recent meta-analysis of 18 studies gave similar results for the evaluation of ventricular arrhythmia with CMR; the prevalence of SHD was 39% overall, 24% in the PVC and NSVT subgroup, and 63% in the complex arrhythmia subgroup (10). The main anomaly was nonischemic cardiomyopathy (56%) (10). CMR is the only imaging modality capable of noninvasively detecting the presence and extent of fibrosis. In our cohort, we found that the most predominant type of structural anomaly (regardless of the presence or absence of known SHD) was nonischemic fibrosis. The presence of nonischemic fibrosis is known to be associated with a risk of serious ventricular arrhythmia. Hammersley et al. reported that the presence of nonischemic fibrosis was a predictor of ventricular arrhythmia and SCD, and the quantification of nonischemic fibrosis enabled risk stratification independently of the LVEF (11). In a study of 4,031 SCD victims in Finland, 24% had a nonischemic cause and 3.6% had myocardial fibrosis without a SHD identified at autopsy and without a channelopathy. 27% of the SCD victims had pathogenic genetic variants associated with cardiomyopathies like ARVD, HCM, or DCM. These variants were particularly frequent in individuals under the age of 40, which suggests that fibrosis is an early, variable, phenotypic expression of these cardiomyopathies (12). LGE (particularly in the epicardial or mid-myocardial layers) is associated with the occurrence of malignant ventricular arrhythmia and SCD in high-level athletes (13) and in patients under the age of 40 (14).

The second most frequently observed structural anomaly in our population was ischemic fibrosis. After a myocardial infarction, the nonexcitable scar tissue surrounded by viable myocytes leads to the creation of heterogeneous conduction zones, which are responsible for ventricular arrhythmia (15). Quantification of the extent of lesions with CMR might help predict the risk of ventricular arrhythmia and thus better identify patients who would benefit from an implantable defibrillator (ICD) (16–18).

A recent study showed that CMR can identify prognostic factors for fatal ventricular arrhythmia, regardless of whether patients had a known SHD (19). The presence of fibrosis is useful for risk stratification in both ischemic (20) and nonischemic (4, 21) disease. In the context of sarcomeric HCM, the American guidelines recommend measuring the extent of fibrosis as a guide to the indication for an ICD for primary prevention (22, 23).

### Relationships between structural abnormalities detected by CMR and the origin of PVCs

The link between structural abnormalities on CMR and the origin of PVCs is difficult to establish, and published data are scarce. The distribution of nonischemic LGE might help to identify the substrate for inducible ventricular arrhythmia during ventricular stimulation in patients with an LVEF < 50%, since there might be an association between nonischemic fibrosis zones and the origin of ventricular arrhythmias (24).

CMR can identify areas of scar tissue, which are sometimes difficult to map with other techniques (25). Even though some PVC patterns do not seem to correlate with the location of abnormalities on CMR, one cannot rule out the presence of other types of PVCs that cannot be detected on ECG. It would be beneficial for all patients to undergo a 12-lead Holter ECG.

In our study, the presence of PVCs with various patterns was predictive of a structural anomaly on CMR. For several nonspecific abnormalities (e.g., diffuse fibrosis, dilation, and ventricular dysfunction), mapping is essential for establishing a link between the PVC pattern and the anatomical origin.

Some nonspecific structural RV abnormalities provide information on the risk of arrhythmia. In a study of 396 patients with >1000 PVCs/24 h, an LBBB pattern, inferior axis deviation, an unremarkable medical history, and normal functional testing and ECG results, RV abnormalities were found in 126 (31.8%) cases and were associated with a significantly elevated risk of cardiac death, resuscitated cardiac arrest, or an appropriate ICD shock. Only 6 of the 126 patients were ultimately diagnosed with ARVD, based on the European Task Force consensus criteria (26). These findings suggest that nonspecific RV abnormalities or suspected ARVD on CMR – even when not confirmed by the European Task Force criteria – require close monitoring and might warrant CMR follow-up. According to Macia et al., 20% of CMR datasets acquired before PVC ablation in patients with an infundibular pattern showed early features of ARVD (27).

Even though the prognosis of PVCs in patients without underlying known SHD is similar to that observed in the general population (1), the absence of SHD does not mean that PVCs are benign; other unidentified conditions (e.g., channelopathies causing Brugada syndrome, long QT syndrome, short QT syndrome, and catecholaminergic polymorphic VT) may be responsible for severe ventricular arrhythmias and SCD (28).

### Predictive factors for CMR abnormalities

Our results are consistent with the literature on the associations between age, male sex, PPVCs, the absence of PVC reduction with exercise (8, 29–32) and the presence of structural abnormalities on CMR. The European Society of Cardiology's 2022 guidelines on ventricular arrhythmias (2) are not clear with regard to the concept of “advanced age”, and so we determined an age threshold (61 years) beyond which CMR provides significant added value. This age threshold is in line with the report by Hosseini et al. in which patients over the age of 60 with at least 5% PVCs but no known SHD or abnormal findings on TTE had a higher risk of structural abnormalities on CMR (8).

The PVCs’ behavior during exercise appears to be directly related to the presence of a structural anomaly. In our study, an increase in the frequency of PVCs during exercise and during recovery was not significantly associated with the detection of a structural anomaly. However, two recent studies found that exercise-induced PVCs in athletes were strongly associated with the presence of LGE (33), and that an increase in PVCs during exercise and recovery was associated with higher all-cause mortality (34). This discrepancy may be related to 67.5% of the patients in our study were taking antiarrhythmic drugs. Furthermore, the increase in PVCs during exercise is associated with higher mortality, regardless of the ischemic changes seen on the exercise test (35). In contrast, the decrease in PVCs with exercise was significantly associated with the absence of CMR findings.

The PVC burden does not appear to predict the discovery of structural abnormalities on CMR (4, 36),, perhaps because it is biased by the use of antiarrhythmic drugs.

### Factors predictive of normal CMR findings

We found that five variables (age <61, the absence of diabetes, the absence of known SHD, normal TTE findings, and monomorphic PVCs that decrease in frequency during exercise) were predictive of normal CMR findings. Patients meeting all these criteria might not need to undergo CMR.

To the best of our knowledge, there is no literature data on factors associated with normal CMR findings in patients with PVCs. Further prospective studies are needed to confirm our present findings and to determine whether these criteria can reliably guide clinical decision-making.

### Follow-up data

We present limited follow-up data due to a short study time. One patient died during data collection from a non-cardiac cause; 12 patients underwent ablation for symptomatic ventricular hyperexcitability despite medical treatment, regardless of CMR results; and 2 patients received implantable cardioverter-defibrillators for primary prevention following CMR findings (one patient with a non-ischemic focal fibrosis, and the other a non-viable necrosis in the context of non-systolic ventricular tachycardia).

### Practical implications

Our results show that CMR can be of value in patients with PVCs, whether or not they have known SHD. CMR is particularly valuable in older (over-61) men with diabetes and PPVCs that do not decrease in frequency during exercise.

A stress test is not included in the European guidelines (2) but provides important information on the PVC patterns. When feasible, a stress test should be part of the initial workup, along with TTE. A decrease in the frequency of PVCs with exercise suggests that there are no structural abnormalities, whereas the persistence of PVCs or an increase in frequency should trigger additional CMR investigations.

### Perspectives

CMR is also valuable prior to PVC ablation. Indeed, a zone of fibrosis is identified in more than a third of cases (37). In both ischemic and nonischemic heart diseases, the analysis and quantification of scar tissue zones are valuable before an ablation procedure because as LGE areas can be integrated into mapping and help to define the ablation strategy (38–41).

### Study limitations

The main limitation of the present study was its retrospective nature, even though consecutive patients were recruited. Prospective studies are needed to confirm our results. Secondly, we did not assess the presence of interstitial fibrosis through measurement of the extracellular volume. Thirdly, CMR was requested by the cardiologists treating the patients with PVCs; hence, the CMR findings might have been influenced by selection bias and might not be representative of all patients with PVCs. Another limitation of this study is the potential risk of overfitting related to the construction of the multivariable model. Although an initial exploratory model included all candidate variables, the final model was reduced to a limited number of variables in order to improve robustness and comply with the recommended events-per-variable ratio. However, no formal internal validation procedure (such as bootstrapping or cross-validation) was performed, which may limit the generalizability of the findings. The ROC analysis was performed on the same cohort used for model derivation, without internal validation by bootstrapping or cross-validation and without validation in an external cohort. Therefore, the model should be considered exploratory and requires external validation before clinical implementation.

## Conclusion

CMR is a major diagnostic tool in the assessment of PVCs and enables the detection of structural abnormalities in nearly half of a population of nonselected patients. CMR is particularly valuable in diabetic patients over the age of 61 with known cardiomyopathy or complex PVCs. Conversely, young patients with no comorbidities, a normal echocardiography and monomorphic PVCs that decrease in frequency with exercise have normal CMR findings, raising the hypothesis that the usefulness of CMR in this specific population should be discussed. A prospective study is needed to validate our data and evaluate the prognostic impact of CMR.

## Data Availability

The original contributions presented in the study are included in the article/Supplementary Material, further inquiries can be directed to the corresponding author.
